# Cardiometabolic multimorbidity (CMM) among older adults in India

**DOI:** 10.1186/s12872-025-05190-w

**Published:** 2025-10-21

**Authors:** Manish Barik, Sushree Nibedita Panda

**Affiliations:** 1 IMPACT Global Consulting, New Delhi, India; 2 The George Institute for Global Health, Hyderabad, India

**Keywords:** Cardiometabolic multimorbidity, Older adults, Ageing, LASI: india

## Abstract

**Background:**

Cardiometabolic multimorbidity (CMM), the co-occurrence of two or more cardiometabolic conditions, poses a growing health concern as populations age. Linked to adverse outcomes like cognitive decline, poor COVID-19 prognosis, and higher mortality, its prevalence is rising due to shared risk factors among conditions such as diabetes, heart disease, and hypertension. While CMM is well-studied in high-income countries, data from low- and middle-income countries, particularly from India, is limited. This study investigates the prevalence and correlates of CMM among older Indian adults.

**Methods:**

We analyzed data from 59,764 participants aged 45 years and older from the Longitudinal Ageing Study in India (LASI), wave 1, conducted from 2017 to 2019. CMM was defined as the co-occurrence of two or more of the following conditions: hypertension, diabetes, coronary heart disease, stroke, obesity, and hypercholesterolemia. Descriptive statistics were employed to calculate the prevalence of CMM with 95% confidence intervals to predict uncertainty. Multivariable logistic regression was used to examine the associations between CMM and various socio-demographic correlates, with results reported as adjusted odds ratios (AOR).

**Results:**

We observed higher CMM prevalence among females 27.3% (95% CI: 26.8%-27.8%) compared to males 23.04% (95% CI: 22.5%-23.5%), particularly in the 60–74 age group. CMM was more prevalent in urban areas, wealthier socioeconomic groups, and those with higher education. Increased odds of CMM were found among males from the OBC and other castes and females from SC, OBC, and “Others” castes, while ST males had lower odds. Significant correlates for both genders included not working, urban residence, and higher wealth. Regionally, CMM rates were highest in the Southern and Western regions, and lowest in the North-East and Central regions of India.

**Conclusion:**

This study reveals a rising burden of CMM among middle-aged and older adults in India, with higher prevalence in females, urban residents, and wealthier groups. Regional and caste disparities highlight the need for targeted interventions. Effective management of CMM requires early screening and comprehensive primary care, especially as India ages and develops.

## Introduction

Cardiometabolic multimorbidity, characterized by the simultaneous presence of two or more cardiometabolic conditions, has become a significant public health concern, particularly as the population ages. Recent data indicates that 4.7% of individuals aged 60 and older are affected by at least two coexisting cardiometabolic diseases [1–3]. Evidence has shown that CMM is linked to a higher risk of various adverse outcomes, including cognitive impairment, depression, increased susceptibility to infections, poor COVID-19 prognosis, and elevated all-cause mortality [2–6].

At age 60, individuals with a history of concomitant diabetes, myocardial infarction, and stroke experience a significant reduction in life expectancy, with a decrease of 15 years compared to those without these conditions [7]. Given the global trend of an aging population, it is not surprising that CMM is becoming increasingly prevalent. This rise is attributable to the fact that many cardiometabolic conditions, such as coronary heart disease (CHD), stroke, diabetes, and hypertension, share overlapping risk factors, similar etiologies, and bidirectional interactions [5–8]. For instance, diabetes or hyperglycemia is a major risk factor for atherosclerotic diseases, including CHD, cerebrovascular disease, and peripheral arterial disease [9]. Moreover, the life-course perspective underscores that cardiometabolic conditions emerge from long‐standing exposures to environmental, behavioral, and socioeconomic influences [10]. In low-and middle income countries (LMICs) and India, the number of people experiencing multimorbidity has increased rapidly over the past few decades [11–13]. Although several studies have been conducted in high-income countries (HICs) on the burden and effect of cardio-metabolic multimorbidity, this topic remains an emerging research area of research in LMICs [6, 14]. Despite increasing recognition of CMM as a key determinant of health outcomes, significant gaps persist in understanding its drivers and consequences in diverse sociocultural settings. In India, where health systems are typically organized around single‐disease vertical programs, CMM often falls through the cracks of care delivery. Recently, a few studies in India have examined the prevalence of multimorbidity across various populations [15–18]. However, there is limited information on the prevalence and trends of CMM specifically among older adults in India. This study aims to address this gap by investigating the prevalence of CMM in Indian older adults and identifying the correlates of this condition using nationally representative population-based data.

## Methods

### Overview of data

This study utilized data from the first wave of the Longitudinal Ageing Study in India (LASI), conducted during 2017–18. LASI is a national survey designed to systematically examine the health, economic conditions, social determinants, and consequences of population aging in India. The initial wave included a baseline sample of 72,250 participants aged 45 years and older, as well as their partners, regardless of age, from all states and union territories of India, with the exception of Sikkim.

To obtain the final unit of observation, LASI employed a multi-stage stratified cluster sampling approach, utilizing three stages of sampling for rural areas and four stages for urban areas. Comprehensive details regarding the sample design, survey instruments, data collection methods, response rates, and fieldwork procedures are available in the LASI report, which can be accessed on their website [19]. In LASI, a total of 72,250 participants were surveyed. We included 59,764 participants aged ≥ 45 years whose complete data on individual as well as biomarkers were available after excluding incomplete/missing data.

### CMM ascertainment

CMM was defined as the co-occurrence of two or more of the following conditions: hypertension, diabetes, coronary heart disease, stroke, obesity, and hypercholesterolemia [20]. We used six self-reported chronic illnesses (hypertension, diabetes, chronic heart disease, stroke, hypercholesterolemia and obesity to create CMM as a simple count of all conditions in an individual where each condition was scored one.

## Individual characteristics

We used age, sex (male/female), residence, education, caste, region, working status, partner status, and wealth index among individual characteristics. For this study, participants’ age, a continuous variable was categorized as ’45–59 years’, ‘60–74 years’, and ‘75 years and above’. Residence of the participants were classified as ‘rural’ and ‘urban’. Education was classified as having ‘formal education’ (those who ever attended school) and ‘no formal education’ (those who never attended school). Caste was labelled as ‘scheduled caste (SC)’, ‘scheduled tribe (ST)’, ‘other backward class (OBC)’; general and no caste were clubbed as ‘others’. States were grouped on the basis of their geographic location as ‘North’, ‘Central’, ‘East’, ‘North-East’, ‘West’ and ‘South’ categorized as region. Working status was segregated as ‘currently working’; and those who currently did not work or had never worked in their lifetime were combined under ‘currently not working’. Participants in live-in relationships and currently married were allocated to ‘living with partner’ and those who were separated, divorced, widowed, never married and deserted were grouped as ‘living without partner’. The economic status of the participants was classified as poorest, poorer, middle, richer and richest based on the monthly per capita expenditure (MPCE).

## Personal/behavioural attributes

Alcohol intake among participants was categorized as ‘yes’ and ‘no’. Tobacco consumption was categorized on the basis of the type of tobacco used i.e. ‘smokeless tobacco’, ‘smoking’, both smoke and smokeless tobacco ‘dual use’ and ‘none’.

## Health attributes

Mean arterial pressure was formulated as 2/3 diastolic pressure + 1/3 systolic pressure. The cut-off for hypertension was fixed as systolic: >140 mm Hg and diastolic: >90 mm Hg classified as ‘hypertensive’ and ‘non hypertensive’. Obesity was categorized as ‘non-obese’ and ‘obese’, based on body mass index (BMI) ≥ 25 kg/m^2^ [21].

### Statistical analysis

Data were analyzed using STATA version 16.0 (STATA Corp., Texas). Descriptive statistics were employed to report the frequency and proportions of socio-demographic characteristics and the period prevalence of CMM. Cases with missing values were excluded from the analysis. Binary logistic regression assessed the relationship between participant characteristics and CMM. Statistically significant variables (*p* < 0.05) from the unadjusted model were included in a multivariable logistic regression to determine associations between CMM and various factors, reported as adjusted odds ratios (AOR) with 95% confidence intervals (CI). To assess model robustness, we conducted post-estimation diagnostics. Variance Inflation Factors (VIFs) were calculated for all covariates. Sampling weights were applied in both descriptive and regression analyses, with 95% CI used to represent uncertainty in weighted proportions.

## Ethical considerations

Ethical approval for LASI was granted by the Indian Council of Medical Research (ICMR) and the International Institute of Population Sciences, Mumbai. LASI obtained informed written consent from all participants. This study uses secondary anonymous data from LASI, posing no risk to participants. All data sources are properly acknowledged and cited.

## Results

This study included 59,764 participants, of whom 53.7% were female, with ages ranging from 45 to 116 years. Nearly two-thirds of the participants resided in rural areas. The majority of both male and female participants were aged between 45 and 59. Most participants lived with their partners. Alcohol consumption was reported by approximately 30% of the male participants, compared to only 2.6% of females. Table 1.

**Table 1:** Socio-demographic characteristics of the study participants

Socio-demographic characteristics	Categories	Male *n* (%)	Female *n* (%)
Age (years) *n* = 59,764	45–59 years	13,173(48.1%)	16,582(51.2%)
	60–74 years	11,072(40.4%)	12,466(38.5%)
	75 years and above	3,159(11.5%)	3,310(10.2%)
Education *n* = 59,763	Educated	18,201(66.4%)	11,187(34.6%)
	No formal education	9,203(33.6%)	27,171(65.4%)
Caste *n* = 59,275	SC	5,263(19.3%)	6,284(19.6%)
	ST	2,302(8.4%)	2,810(8.7%)
	OBC	12,510(45.9%)	14,652(45.7%)
	Others	7,134(26.2%)	8,317(25.9%)
Residence *n* = 59,764	Rural	19,493(71.1%)	22,265(68.8%)
	Urban	7,912(28.8%)	10,093(31.2%)
Region *n* = 59,764	North	2,107(7.6%)	2,541(7.9%)
	Central	7,426(27.1%)	8,035(24.8%)
	East	6,730(24.6%)	7,449(23.0%)
	North-East	974(3.6%)	1,080(3.3%)
	West	4,161(15.2%)	5,291(16.4%)
	South	6,004(21.9%)	7,963(24.6%)
Working status *n* = 59,760	Currently working	18,137(66.2%)	9,788(30.3%)
	Current not working	9,266(33.8%)	22,568(69.8%)
Partner status *n* = 59,058	With partner(live-in relationship, currently married)	24,015(88.8%)	20,294(63.4%)
	Without partner (separated/divorced/widowed/never married/deserted)	3,019(11.2%)	11,729(36.6%)
Wealth Index *n* = 59,764	Poorest	5,712(20.8%)	6,903(21.3%)
	Poorer	5,850(21.4%)	6,864(21.2%)
	Middle	5,593(20.4%)	6,616(20.5%)
	Richer	5,361(19.6%)	6,363(19.7%)
	Richest	4,887(17.8%)	5,612(17.3%)
**Personal/Health Behaviour**			
Alcohol consumption *n* = 59,701	Yes	8,219(30.0%)	844(2.6%)
	No	19,143(69.9%)	31,494(97.4%)
Tobacco consumption *n* = 59,682	Smoke	7,496(27.4%)	1,058(3.3%)
	Smokeless	6,996(25.6%)	5,165(15.9%)
	Dual	1,715(6.3%)	114(0.4%)
	Never Used	11,147(40.8%)	25,988(80.4%)

In Table 2, the prevalence of CMM was found to be higher among females 27.3%(95% CI 22.5%-23.5%) compared to males 23.04%(95% CI 26.8%-27.8%). The 60–74 years age group exhibited the highest CMM prevalence across both genders. Participants with formal education reported elevated CMM prevalence (28.1% in males and 40.1% in females), and those residing in urban areas (39.5% in males and 44.3% in females) has the highest prevalence while rural participants demonstrated lower prevalence. Older adults from wealthier socioeconomic categories had higher CMM prevalence compared to those in the poorest groups, irrespective of gender. Participants who were not currently employed (29.7% in males and 27.7% in females) or living without a partner (23.9% in males and 27.5% in females) also showed increased CMM prevalence. Furthermore, regional disparities were observed, with the Southern and Western regions exhibiting higher CMM rates, whereas the North-East and Central regions had lower prevalence for both males and females.

**Table 2:** Prevalence of CMM across various individual attributes

Socio-demographic characteristics	Categories	Prevalence of Cardio-metabolic multimorbidity (CMM)			
		Male CMM prevalence 23.04% (22.5%−23.5%)		Female CMM prevalence 27.3% (26.8%−27.8%)	
		%	95% CI	%	95% CI
Age (years) *n* = 59,764	45–59 years	21.6%	20.9%−22.3%	25.7%	25.1%−26.4%
	60–74 years	25.2%	24.4%−26.1%	30.7%	29.8%−31.5%
	75 years and above	21.2%	19.6%−22.4%	22.3%	20.9%−23.8%
Education *n* = 59,763	Educated	28.1%	27.4%−28.7%	40.7%	39.8%−41.6%
	No formal education	13.1%	12.3%−13.7%	20.2%	19.6%−20.7%
Caste *n* = 59,275	SC	16%	15%−17%	21.3%	20.3%−22.3%
	ST	11%	9.6%−12.2%	10.6%	9.5%−11.8%
	OBC	25.1%	24.2%−25.8%	28.5%	27.8%−29.3%
	Others	28.6%	27.5%−29.6%	35.1%	34.1%−36.2%
Residence *n* = 59,764	Rural	16.3%	15.8%−16.8%	19.6%	19.1%−20.1%
	Urban	39.5%	38.4%−40.6%	44.3%	43.3%−45.3%
Region *n* = 59,764	North	31.2%	29.2%−33.2%	38.2%	36.3%−40.1%
	Central	14.2%	13.4%−15.1%	18%	17.1%−18.8%
	East	17.5%	16.6%−18.5%	20.1%	19.1%−20.9%
	North-East	16.8%	14.5%−19.2%	17%	14.7%−19.2%
	West	29.7%	28.3%−31.1%	32.8%	31.5%−34.1%
	South	33.5%	32.3%−34.7%	37.7%	36.6%−38.8%
Working status *n* = 59,760	Currently working	20%	19.3%−20.5%	17.4%	16.6%−18.2%
	Current not working	29.7%	28.7%−30.6%	27.7%	26.7%−28.8%
Partner status *n* = 59,058	Living with partner	23.9%	23.3%−24.4%	27.5%	26.8%−28.1%
	Living without partner	17.7%	16.3%−19.1%	27.3%	26.5%−28.2%
Wealth Index *n* = 59,764	Poorest	15.3%	14.4%−16.3%	18.7%	17.7%−19.6%
	Poorer	19.1%	18.1%−20.1%	22.1%	20.7%−22.6%
	Middle	21.8%	20.7%−22.9%	26.8%	25.75–27.8%
	Richer	25.1%	23.8%−26.2%	32.3%	31.1%−33.5%
	Richest	35.9%	34.6%−37.3%	39.2%	37.9%−40.5%
	Personal/Health Behaviour				
Alcohol consumption *n* = 59,701	Yes	19.8%	18.9%−20.6%	12.1%	9.8%−14.3%
	No	24.4%	23.8%−25.1%	27.7%	27.2%−28.2%
Tobacco consumption *n* = 59,682	Smoke	18.1%	17.2%−19.1%	11.9%	9.9%−13.9%
	Smokeless	17.6%	16.7%−18.5%	20.1%	18.9%−21.1%
	Dual	14.6%	13.1%−16.4%	13.4%	7.6%−20.9%
	Never Used	31.1%	30.1%−31.8%	29.4%	28.8%−30%

In Table 3, for males, those aged 60–74 years demonstrated a significantly higher likelihood of developing CMM [AOR: 1.20 (1.04–1.38)]. Increased CMM prevalence was also associated with higher education [AOR: 1.79 (1.55–2.07)] and urban residence [AOR: 2.42 (2.07–2.82)]. Males from the OBC caste [AOR: 1.23 (1.05–1.43)] and “Others” caste [AOR: 1.30 (1.11–1.51)] had increased odds of CMM, whereas those from the ST caste [AOR: 0.76 (0.62–0.94)] had reduced odds. Not currently working [AOR: 1.72 (1.51–1.97)] and higher wealth [AOR: 2.51 (1.91–3.31)] were also significant correlates s of increased CMM risk. Regionally, males from the Southern [AOR: 2.23 (1.83–2.71)] and Western [AOR: 1.91 (1.63–2.25)] regions had the highest likelihood of developing CMM.

For females, similar trends were observed, with those aged 60–74 years showing elevated CMM odds [AOR: 1.31 (1.16–1.48)]. Higher education [AOR: 1.59 (1.40–1.80)] and urban residence [AOR: 2.17 (1.92–2.46)] were also linked to increased CMM prevalence. Females from SC, OBC, and “Others” castes had higher CMM odds, with the “Others” caste showing the highest increase [AOR: 2.23 (1.78–2.80)]. Wealthier females and those not working [AOR: 1.67 (1.47–1.90)] had higher CMM risks, while smokeless tobacco use was significantly associated with increased CMM odds [AOR: 1.66 (1.27–2.18)]. Dual use was not a significant associated factor for CMM in females. Table 3.

**Table 3:** Multivariable regression analysis depicting association of CMM with various socio-demographic attributes

Socio-demographic characteristics	Categories				
		Male		Female	
		AOR	95%CI	AOR	95.%CI
Age (years) *n* = 59,764	45–59 years	Ref		Ref	
	60–74 years	1.20	1.04–1.38**	1.31	1.16–1.48**
	75 years and above	0.79	0.61–1.02	0.88	0.71–1.09
Education *n* = 59,763	Educated	1.79	1.55–2.07**	1.59	1.40–1.80**
	No formal education	Ref		Ref	
Caste *n* = 59,275	SC	Ref		1.85	1.46–2.35**
	ST	0.76	0.62–0.94*	Ref	
	OBC	1.23	1.05–1.43*	1.84	1.47–2.30**
	Others	1.30	1.11–1.51**	2.23	1.78–2.80**
Residence *n* = 59,764	Rural	Ref			
	Urban	2.42	2.07–2.82**	2.17	1.92–2.46**
Region *n* = 59,764	North	1.99	1.71–2.32**	1.94	1.70–2.22**
	Central	Ref		Ref	
	East	1.38	1.18–1.62**	1.13	0.98–1.31
	North-East	1.27	1.06–1.53**	0.98	0.82–1.17
	West	1.91	1.63–2.25**	1.75	1.51–2.03**
	South	2.23	1.83–2.71**	2.14	1.82–2.52**
Working status *n* = 59,760	Currently working	Ref		Ref	
	Current not working	1.72	1.51–1.97**	1.67	1.47–1.90**
Partner status *n* = 59,058	Living with partner	Ref		Ref	
	Living without partner	0.71	0.60–0.84	0.95	0.83–1.08
Wealth Index *n* = 59,764	Poorest	ref			
	Poorer	1.32	1.09–1.60**	1.17	0.99–1.39
	Middle	1.47	1.20–1.79**	1.43	1.19–1.72**
	Richer	1.67	1.39–2.01**	1.79	1.49–2.15**
	Richest	2.51	1.91–3.31**	2.17	1.79–2.64**
Alcohol consumption *n* = 59,701	Yes	Ref		Ref	
	No	1.00	0.88–1.14	1.33	0.99–1.81
Tobacco consumption *n* = 59,682	Smoke	1.00	0.79–1.25	Ref	
	Smokeless	1.14	0.92–1.41	1.66	1.27–2.18**
	Dual	Ref		1.46	0.75–2.83
	Never Used	1.52	1.24–1.87**	1.96	1.54–2.51**

Ref: Reference

** *p* < 0.001, * *p* < 0.05

## Discussion

In this study of 59,764 participants (53.7% female, aged 45–116) we found higher CMM prevalence among females (27.3%) than males (23.04%), particularly in the 60–74 age group. CMM was more common among urban residents, wealthier socioeconomic groups, and those with higher education. Males from the OBC and “Others” castes and females from SC, OBC, and “Others” had increased CMM odds, while ST males had reduced odds. Not working, urban residence, and wealthier status were significant correlates s for both genders. Regionally, the Southern and Western regions exhibited higher CMM rates, while the North-East and Central regions had lower prevalence. This information is not only critical for targeting of appropriate interventions or prevention strategies to reach those most in need; they also imply that the prevalence of multimorbidity will rise in the future as India’s population ages and continues to undergo rapid economic development and urbanization [22].

The CMM prevalence estimated by this study was 27.3% among middle-aged and older Indian women, previous studies conducted on the same population has also found the overall multimorbidity to 29.7%, similar finding emerged in the study conducted on Chinese women which stated 33.1% [20, 23]. These data suggest that India is undergoing a growing cardiometabolic disease epidemic, likely driven by shifts in dietary patterns and lifestyle behaviours due to rapid demographic and socioeconomic transitions [24]. Studies also indicate a high prevalence of cardiometabolic syndromes in Western countries and other parts of Asia, such as the United States (35%), Iran (37%), and Turkey (44%) [6, 25, 26]. Differences in prevalence between LMICs and HICs may reflect actual variation or differences in definitions and sampling methodologies [27].

The results from this research also suggested that CMM increases with age and is more prevalent among individuals residing in urban areas, wealthier socioeconomic strata, and those who are employed, compared to their rural and non-working counterparts. Consistent with previous research, the burden of cardiometabolic disease escalates with age, likely due to the progressive decline in cardiometabolic function [26, 28]. The higher prevalence of CMM among urban, wealthier, and employed individuals may be attributable to lifestyle factors such as physical inactivity, sedentary behaviour, and unhealthy dietary habits [29, 30], including the excessive consumption of high-calorie foods, fats, and salt, associated with urbanization and economic development [31, 32].

In addition, there was increased odds of risk CMM was seen among the educated older adults which is another important indicator of socioeconomic status [33]. An unexpected finding was the negative association between current smoking and CMM in males. This may be explained by a negative association between smoking and obesity (a risk factor for diabetes and hypertension), or it could be due to individuals with chronic conditions being more likely to quit or underreport smoking.

### Study implications

To strengthen early detection of high-risk groups for CMM, enhanced screening and targeted preventive strategies are crucial. A robust, people-centered primary care system should prioritize cardiometabolic diseases and multimorbidity, as this has proven to be the most cost-effective way to manage chronic conditions [34–36]. An effective approach to managing CMM involves shifting care from specialists to primary care, enabling early detection and integrated management of comorbid conditions—especially in resource-limited settings like India. Our findings point to a high burden of coexisting cardiometabolic conditions among older Indian adults, underscoring the relevance of “integrated care approaches”. While we did not evaluate models of care in this study, such as “one-stop clinics” or community-based chronic disease management strategies, these approaches are increasingly emphasized in public health policy for multimorbidity globally. Their relevance is supported by the clustering of conditions observed in our sample and by recent calls for strengthened primary care responses in India and globally. State health systems should pilot “one-stop” CMM clinics within district hospitals, staffed by multidisciplinary teams (e.g., physicians, dietitians, physiotherapists) to manage interconnected conditions. The Ministry of Health should revise the National NCD guidelines to include multimorbidity management, such as prioritizing high-impact interventions, addressing polypharmacy, and supporting de-prescribing. Incorporating CMM principles into medical and nursing curricula will strengthen future health workforce capacity. Additionally, equity-focused monitoring should disaggregate CMM indicators by gender, caste, socioeconomic status, and geography. Tools like community scorecards and participatory feedback loops can also ensure programs remain inclusive, locally relevant, and responsive to diverse needs.

### Strength and limitations

To the best of our knowledge, this is the first nationally representative study focused on older adults that explores gender differences in cardiometabolic multimorbidity, while also examining rural-urban disparities and related factors using longitudinal data and cardiometabolic biomarkers for disease diagnosis. However, the study has some limitations. First, although several cardiometabolic diseases were identified using biomarkers, the reliance on self-reported data for heart disease and stroke may have led to an underestimation of their prevalence. Second, the prevalence of cardiometabolic multimorbidity was estimated by simply counting chronic conditions. While we recognize that factors such as diet, physical activity, medication use, and family history are important determinants of cardiometabolic health, these variables were not consistently available in the LASI Wave 1 dataset and could not be reliably included in our analysis. Similarly, although interaction analyses (e.g., gender × education or urban × wealth) could provide additional insights into effect modification, the primary objective of this study was to describe the overall prevalence and social patterning of CMM. Incorporating complex interaction terms would have increased model complexity and reduced interpretability in this large and heterogeneous sample. Future research could explore these dimensions using more detailed or longitudinal data.

## Conclusion

This study highlights the growing burden of CMM among middle-aged and older adults in India, particularly among females, urban residents, and wealthier individuals. Regional and caste disparities further emphasize the need for targeted interventions. Early screening and comprehensive primary care are essential for managing CMM, especially as India’s population ages and economic development accelerates.

## Data Availability

The dataset analysed during the current study is available in the LASI data repository held at ICT, IIPS [https://g2aging.org/?section=overviews&study=lasi].

## Abbreviations

LASI: Longitudinal Ageing Study India
LMICs: Low-and-middle Income Countries
DALY: Disability-Adjusted Life Years
HICs: High-Income Countries
MPCE: Monthly per Capita Expenditure
